# A proteomic analysis of *Curcuma comosa *Roxb. rhizomes

**DOI:** 10.1186/1477-5956-9-43

**Published:** 2011-07-29

**Authors:** Apaporn Boonmee, Chantragan Srisomsap, Daranee Chokchaichamnankit, Aphichart Karnchanatat, Polkit Sangvanich

**Affiliations:** 1 Department of Chemistry, Faculty of Science, Chulalongkorn University, Bangkok, 10330, Thailand; 2 Laboratory of Biochemistry, Chulabhorn Research Institute, Bangkok, 10210, Thailand; 3 Research Institute of Biotechnology and Genetic Engineering, Chulalongkorn University, Bangkok, 10330, Thailand

**Keywords:** Curcuma comosa Roxb, Proteomic, MicroSol-IEF, Zoom-IEF, Lectin, Superoxide dismutase

## Abstract

**Background:**

The similarly in plant physiology and the difficulty of plant classification, in some medicinal plant species, especially plants of the Zingiberaceae family, are a major problem for pharmacologists, leading to mistaken use. To overcome this problem, the proteomic base method was used to study protein profiles of the plant model, Curcuma comosa Roxb., which is a member of the Zingiberaceae and has been used in traditional Thai medicine as an anti-inflammatory agent for the treatment of postpartum uterine bleeding.

**Results:**

Due to the complexity of protein extraction from this plant, microscale solution-phase isoelectric focusing (MicroSol-IEF) was used to enrich and improve the separation of Curcuma comosa rhizomes phenol-soluble proteins, prior to resolving and analyzing by two-dimensional polyacrylamide gel electrophoresis and identification by tandem mass spectrometry. The protein patterns showed a high abundance of protein spots in the acidic range, including three lectin proteins. The metabolic and defense enzymes, such as superoxide dismutase (SOD) and ascorbate peroxidase, that are associated with antioxidant activity, were mainly found in the basic region. Furthermore, cysteine protease was found in this plant, as had been previously reported in other Zingiberaceae plants.

**Conclusion:**

This report presents the protein profiles of the ginger plant, Curcuma comosa. Several interesting proteins were identified in this plant that may be used as a protein marker and aid in identifying plants of the Zingiberaceae family.

## Background

Plants in Zingiberaceae family are widely distributed in many countries of Southeast Asia. In Thailand at least two-hundred species of Zingiberaceous plants are found and these include members of various genera, such as Alpinia, Amomum, Curcuma, Etlingera, Kaempferia, and Zingiber [1]. Zingiberaceous plants have been widely used in traditional medicine, as well as a food flavoring and spice agents. Many studies have focused on the bioactive small organic compounds from these plants and have supported the traditional medicinal use of the plant extracts, such as curcumin [2], sesquiterpene [3-5], and various essential oils [6-8], flavonoids and phenolic compounds [9,10]. In addition, the biologically active proteins reported from Zingiberaceae plants include, antifungal proteins from *Zingiber officinalis *[11] and antioxidant proteins from *C. longa *[12] and *C. zedoaria *[13]. Interestingly, the lectins were also found in many species of this Zingiberaceous plants. The lectins or agglutinin proteins, a class of carbohydrate-binding non-immune origin proteins, have been used as tools in analytical biochemistry [14,15] including in medical applications, such as drug delivery [16], blood typing [17] and potential antineoplastic drugs [18], amongst others. Their actual physiological functions are likely to be in the defense against phytophagous predators (mostly insects) and phytopathogenic microorganisms [19,20]. These plant lectins have been found in a variety of plant species, including the ginger family where, for example, the mannose-binding lectin cDNA, *Z. officinale *agglutinin (ZOA) [21], was cloned from the rhizomes of *Z. officinale*. According to the similarity of DNA sequences between ZOA and two other lectins, that is *Galanthus nivalis *agglutinin (GNA) from the snowdrop, which is highly toxic to sap-sucking insects, and *Gastrodia elata *antifungal protein (GEAFP), belonging to Orchidaceae lectins, ZOA may have defense based activities along the same lines as these two proteins. Heamagglutination activity was previously determined to be present in fifteen *Curcuma *plant species when assaying the crude rhizomal protein extract against rabbit erythrocytes [22], and this array of lectin-like activity positive plants included *C. xanthorhiza*, which is closely related to *C. comosa*. Certainly, purified lectins have been reported in a few Zingiberaceae plants. A 32.4 kDa lectin enriched from *C. amarissima *Roscoe [23] revealed a growth-inhibitory activity against three plant pathogenic fungi (*Fusarium oxysporum*, *Exserohilum turicicum *and *Colectrotrichum cassiicola*), and showed *in vitro *cytotoxicity against the BT474 breast cancer cell line. A thermostable lectin of 41.7 kDa isolated from *Kaempferia parviflora *[24] showed heamagglutination activity against several different erythrocyte sources, with the strongest activity observed against rabbit red blood cells.

However, most plants in this family have very similar botanical characteristics and this makes it very difficult to clearly identify each species. The mistaken identification of medicinal plant materials is a serious problem for both manufacturers of traditional medicine products and researchers. There are a few methods to distinguish each species of plant, such as botanical characteristics by specialized taxonomists or DNA sequence based methods (e.g. establishing molecular operational taxonomic units with conversion to species by sequence identity to known species in the NCBI database). However, although the latter method is tissue and developmental stage independent, it is time consuming and complicated (due to the problem of discrimination of variety/cultivar polymorphism versus cryptic or sibling species).

Recently, proteomic tools have been used to identify types or isolates in many organisms [25-27] so this technique may be the one of the choices for the classification of Zingiberaceous plants. Of course, with no current baseline database it is far from clear how much the proteome for a specific tissue (e.g. rhizome) may vary within a species due to local genetics (cultivars) or cultivation conditions compared to between species, and so how useful this approach could be, but nevertheless under such a scenario it could still be used for following specific cultivars/cultivation conditions for quality control checking of any given cultivar. Thus, the aim of this report was to perform a preliminary study of the phenol-soluble protein profile from *C. comosa *as an initial model plant from the Zingiberaceae family.

Because in traditional Thai medicine, the rhizome is generally the part of the plant that is most wildly use and because a higher amount of protein is present in rhizome than in other parts, the protein database study in bulbous plants and those from *Curcuma *are usually used the rhizomes, respectively. For this reason, we selected *C. comosa*, an herb with large rhizome, as the model Zingiberaceae plant for proteomic study. *C. comosa*, commonly known as Waan Chak Mod Look in Thai, has been used as a traditional medicine for the treatment of postpartum uterine inflammation, perimenopausal bleeding and hemorrhoids. The isolated compounds from this plant have been reported to display various biological properties, such as estrogenic [28], anti-inflammatory [29], choloretic [30], antioxidant [31] and nematocidal [32] activities. However, the protein profile from this plant has not been reported. Therefore, the proteomic analysis of the rhizomes of *C. comosa *is expected to be useful for both establishing the potential of protein fingerprints in Zingiberaceae family and for the investigation of its specific proteins in a high throughput manner.

## Methods

### Protein extraction

Fresh rhizomes of *C. comosa *purchased from a local market in Bangkok, Thailand. A voucher specimen (BKF. No. 97298) is deposited at The Forest Herbarium (BKF), Royal Forest Department, Bangkok, Thailand. Grind fresh tissue of this plant to a powder with liquid nitrogen in a mortar and pestle. Base on *C. longa *proteomic [33], there are some interference compounds need to remove. Therefore the use of selection extraction method and buffer for *C. comosa *was similar with *C. longa *with slightly modification. Briefly, the plant powder (5 g) was extracted by suspension in 20 mL of extraction buffer (0.5 M Tris, 30 mM HCl, 0.1 M KCl, 0.7 M sucrose and 1% (v/v) β-mercaptoethanol) for 30 min at 4°C, whereupon the supernatant was then collected by centrifugation at 4,000 × g for 10 min. The precipitate was extracted twice in extraction buffer and the poled extracts were then extracted with a 1:5 (v/v) ratio of water-saturated phenol at 4°C for 60 min. After phase separation the phenol phase was then harvested and proteins were precipitated from the phenol phase by the addition of a four-volume of 0.1 M ammonium acetate in methanol and left overnight at -20°C. The resulting phenol-soluble protein pellet was collected by centrifugation at 4,000 × g for 10 min, resuspended in cold water with sonication for 3 min and then precipitated again in nine volumes of cold acetone at -20°C for 2 h and centrifuged at 4,000 × g for 10 min. The protein pellet was air-dried to remove the acetone. The amount of protein in each sample was determined by the Bradford assay [34].

### Microscale solution-phase isoelectric focusing (MicroSol-IEF) of the protein extract

Aliquot protein (3 mg) from the isolated proteins (115.5 mg) were dissolved in 0.2 mL of solubilization buffer (7.7 M urea, 2.2 M thiourea and 4.4% (w/v) CHAPS) and then 20 μl of 100 mM iodoacetamide (IAA) was added, mixed and incubated in the dark for 30 min at room temperature. After this incubation, the proteins were then precipitated by the addition of four volumes of cold acetone and harvested by centrifugation, as described above. The protein pellet was resuspended in solubilization buffer and supplemented with 10 mM dithiothreitol (DTT), 0.8% (w/v) ampholine and trace amount of bromophenol blue (The final concentration of protein was approximate 1.5 mg/mL). The Zoom-IEF fractionator (Invitrogen, Carlsbad, CA, USA) was assembled with three disks (pH 3.0, pH 5.4 and pH 10.0). The protein solution (0.65 mL) was loaded between disk pH 3.0-5.4 and pH 5.4-10.0 and focused at 100 V for 20 min, followed by 200 V for 80 min and finally 600 V for 80 min. After separation by Zoom-IEF, the protein solution was kept at 4°C for further analysis.

### Two-dimensional polyacrylamide gel electrophoresis (2-DE)

The protein samples (200 μg) were loaded onto immobilized pH gradient (IPG) gel strips (GE Healthcare, Biosciences, Uppsala, Sweden) and left overnight at room temperature. The first dimension was performed on a Pharmacia LKB Multiphor II system at 7,000 Vh. After electrofocusing, the IPG strips were reduced in equilibration buffer (50 mM Tris-HCl buffer, pH 6.8, 6 M urea, 1% (w/v) sodium dodecyl sulfate (SDS), 30% (v/v) glycerol) containing 1% (w/v) DTT and were alkylated with equilibration buffer containing 2.5% (w/v) IAA. After equilibration, the IPG strips were analyzed in the second-dimension on a SDS polyacrylamide gel (15% (w/v) acrylamide resolving gel) performed in a Hoefer system. Coomassie Brilliant Blue R-250 staining was used to visualize the protein bands.

### Tryptic in-gel digestion

The protein spots were cut out from the gel and the coomassie blue removed using 0.1 M NH_4_HCO_3 _in 50% (v/v) acetonitrile until the gel pieces were colorless. After drying of the gel pieces by Speed Vacuum, the gels were reduced with buffer solution (0.1 M NH_4_HCO_3_, 1 mM Ethylenediaminetetraacetic acid (EDTA) and 10 mM DTT) at 60°C for 45 min. The liquid was removed and then the gel slices covered in 100 mM IAA in 0.1 M NH_4_HCO_3 _solution and incubated at room temperature in the dark for 30 minutes. The residual IAA solution was then removed and the gel pieces were washed with 50% (v/v) acetonitrile (ACN) in water, and dried in a Speed Vacuum. Next a trypsin solution (0.05 M Tris-HCl buffer pH 8.5, 0.1 μg/μL trypsin in 1% (v/v) acetic acid, 10% (v/v) ACN and 1 mM CaCl_2_,) was added to the gel pieces and incubated at 37°C overnight. Thereafter, the solution was collected and the gels were extracted three times with 2% (v/v) trifluoroacetic acid, 0.05 M Tris-HCl buffer pH 8.5 containing 1 mM CaCl_2 _and 2.5% (v/v) formic acid in acetonitrile respectively. The solutions were pooled and dried by Speed Vacuum.

### Protein identification by tandem mass spectrometry

The tryptic peptides were analyzed by using LC/MS/MS, a capillary LC system (Waters) coupled to a Q-TOF mass spectrometer (Micromass, Manchester, UK). The database search was performed with ProteinLynx screening. The Mascot http://www.matrixscience.com/search_form_select.html and the Peaks search tools http://www.bioinfor.com:8080/peaksonline/login.jsp were used for samples where proteins were not found by the ProteinLynx screening. Some proteins were interpreted amino acid sequences using the De novo sequencing tool in Masslynx or the Auto De novo sequencing tool in Peaks online 2.0 and then searched by MS BLAST against the NCBI database http://dove.embl-heidelberg.de/Blast2/msblast.html.

## Results and Discussion

### Sample extraction and 2-D IEF-SDS-PAGE profile

**Figure 1:**
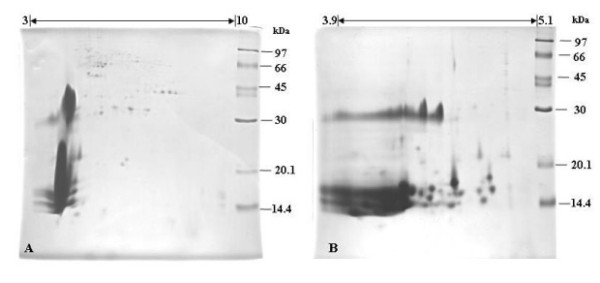
**Two-dimensional (IEF-SDS-PAGE) gel electrophoresis profile of the crude phenol-soluble protein fraction isolated from *C. comosa *rhizomes and analyzed using a linear IEF strip of (A) pH 3-10 and (B) pH 3.9-5.1**. Protein molecular weight markers were co-resolved in the second (SDS-PAGE) dimension on the right hand side, with the sizes indicated in the figure. The gels shown are representative of three such repeats.

**Figure 2:**
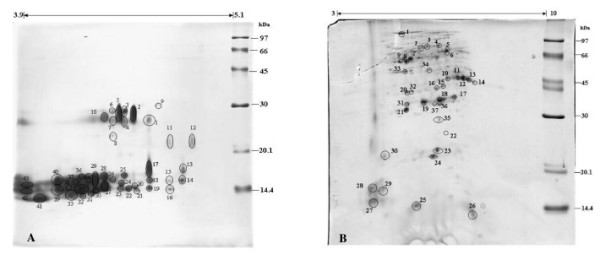
**Two-dimensional (IEF-SDS-PAGE) gel electrophoresis profiles of the crude phenol-soluble protein fraction isolated from *C. comosa *rhizomes after separation and enrichment with microscale solution-phase isoelectric focusing (Zoom IEF) at a pH range of (A) 3-5.4 and (B) 5.4-10**. The two enriched samples were then resolved in the first dimension using a linear IEF strip of (A) pH 3.9-5.1 and (B) pH 3-10. Protein molecular weight markers were co-resolved in the second (SDS-PAGE) dimension on the right hand side, with the sizes indicated in the figure. The gels shown are representative of three such repeats.

The presence of some substances in plant tissues, such as polysaccharides, lipids, lignins, pigments and phenolic compounds, can interfere with the sample preparation for proteomic analysis. To reduce these compounds a phenol extraction followed by methanol/ammonium acetate precipitation was performed in the protein preparation. At the beginning of protein study, the proteins from *C. comosa *rhizomes were run on 2-D IEF-SDS-PAGE using a pH 3 - 10 linear IEF strip (Figure 1A). However, the proteomic pattern showed a high intensity of poorly resolved spots in the acidic region and a low intensity of spots in the basic region, which is somewhat similar to the previously reported protein patterns of *C. longa *[33]. Therefore, to improve the protein separation, a narrow range linear IEF strip of pH 3.9-5.1 was used (Figure 1B). However, even though some proteins in the acidic region were better resolved, it was still difficult to impossible to identify unique spots. To overcome this problem and enrich the low abundance proteins in the basic region, the effective way is to prefractionate sample. There are several techniques for protein prefractionation such as gel chromatography, selective solublization, sub cellular fractionation and isoelectrofocusing (IEF) which is the one of mostly successful due to its highly resolution and compatibility with subsequent 2DE analysis. Recently, microscale solution-phase isoelectric focusing (MicroSol-IEF) [35,36] was developed for protein prefactionation. The commercial device based on this approach is known as ZOOM IEF Fractionator (Invitrogen Corp). The protein will be fractionated and trapped in a multichannel of this chamber depends on their pI values. This technique has been successfully used to separate many types of complexity sample for instance human plasma and serum [37], mouse brain proteins [38] etc. For this reason, we designed to use MicroSol IEF approach to improve protein separation in our study,. The crude proteins were focused in two pH ranges; the acidic region (pH 3-5.4) and the basic region (pH range 5.4-10). The resolution of the protein patterns obtained following 2-D IEF-SDS-PAGE resolution of these two protein ranges were greatly improved (Figure 2).

### Protein Identification

**Figure 3:**
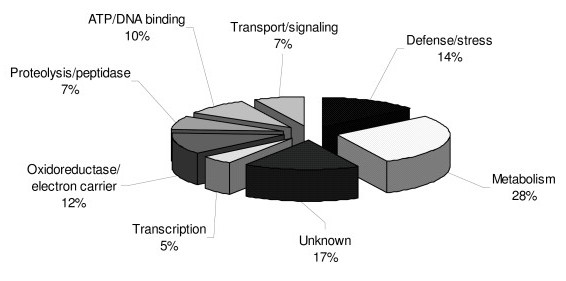
**Functional distribution of the 42 putatively identified phenol-soluble proteins (see Table 1) expressed in *C. comosa *rhizomes**. Protein functions are ascribed from that which was annotated in the database to the likely hit (homolog) found by peptide mapping of the tryptic fragments.

One hundred and eighty-three spots (70 spots from the acidic region and 113 spots from the basic region) were identified with the aid of the ImageMaster 2D Platinum 7.0 software (GE Healthcare Bio-Sciences). The darkest stained eighty spots in the 2D gel pattern (43 from the acidic region and 37 from the basic region, as shown by numbered circles in Figure 2) were chosen for identification by tryptic in-gel digestion and LC/MS/MS. Unfortunately, thirty-eight (23 (~53%) from the acidic region and 15 (~40%) from the basic region) of these 80 spots could not be identified because of the limitations of existing protein sequences in plant protein database. The putatively identified proteins (Table 1) were grouped according to their functions, derived from the annotated function of the homologous protein(s) hits in the database search. Most (jut over one quarter) of the identified proteins were annotated as being involved in metabolic pathways whereas other proteins (in decreasing order of prevalence) were involved in defense/stress response, unknown functions, oxidoreductase/electron carrier, ATP/DNA binding, proteolysis/peptidase, transport/signaling and transcription (Figure 3).

**Table 1:** Phenol-soluble proteins identified from 2-D (IEF-SDS-PAGE) gels of the acidic (pH 3-5.4; spot nos. A2- 40 in figure 2) and basic (pH 5.4-10; spot nos. B4-37 in figure 2) region proteins from *C. comosa *rhizomes, as analyzed by LC/MS/MS.

Spot	Uniport ID	Protein name	Organism	Sequence coverage (%)	Peptides matched	Theoretical		Function
						MW (Da)	pI	
A2	Q40687	Guanine nucleotide-binding protein subunit beta	*Oryza sativa*	3	1	41,726	7.13	Protein signaling
A3	Q9FNA6	Genomic DNA, chromosome 5, P1 clone	*Arabidopsis thaliana*	6	1	60,231	5.56	Unknown
A5	P32033	Protein ycf2	*Cuscuta reflexa*	7	1	234,393	9.25	ATP binding
A8	O64637	Cytochrome P450 76C2	*Arabidopsis thaliana*	3	2	57,221	6.50	Oxidoreductase
A7	Q9MAZ0	Nonclathrin coat protein	*Zea mays*	6	1	19,928	4.81	Protein transport
A9	P30182	DNA topoisomerase 2	*Arabidopsis thaliana*	2	2	164,005	7.25	ATP binding
A11	Q6ZBQ5	Hypothetical protein	*Oryza sativa*	7	1	15,105	11.6	Unknown
A12	Q9ZPH2	Monothiol glutaredoxin-S17	*Arabidopsis thaliana*	3	1	53,082	5.01	Electron carrier
A13	P27898	Myb-related protein P	*Zea mays*	2	1	43,729	10.1	Transcription
A16	A9SAC0	Predicted protein	Physcomitrella patens subsp patens	4	1	17,421.	5.28	Unknown
A17	Q9LDQ8	Similarity to proton pump interactor	*Arabidopsis thaliana*	1	1	58,552	5.66	Unknown
A18	Q2QM00	Type IIB DNA topoisomerase family protein	*Oryza sativa*	1	1	55,342	5.62	DNA/ATP binding
A19	Q9LVP9	Vesicle transport v-SNARE 13	*Arabidopsis thaliana*	4	1	25,026	9.41	Protein transport
A20	Q5I2R0	minus agglutinin (SAD1)	*Chlamydomonas incerta*	1	2	404,525	6.08	Defense
A25	Q9GEZ3	NADH dehydrogenase subunit F	*Gymnosteris parvula*	2	1	37,892	9.74	Oxidoreductase
A27	Q6V8L5	Lectin	*Typhonium divaricatum*	12	4	20,250	9.17	Defense
A31	Q6RHR1	Basic beta-1,3-glucanase	*Capsicum annuum*	6	1	17,521	11.3	Metabolism
A28	O49565	Putative F-box protein At4g21240	*Arabidopsis thaliana*	2	1	48,377	5.83	Unknown
A34	Q41625	Mannose-binding lectin precursor	*Tulipa hybrid cultivar*	13	4	19,556	5.60	Defense
A40	Q0ZIZ4	ATP-dependent Clp protease proteolytic subunit	*Vitis vinifera*	5	1	22,060	4.75	Proteolysis
B4	P93262	Phosphoglucomutase	*Mesembryanthemum crystallinum*	2	1	63,446	5.87	Metabolism
B5	Q94CI8	Glycine-rich protein	*Solanum lycopersicum*	2	1	23,420	5.88	Unknown
B6	Q1PCD2	glucose 6 phosphate isomerase	*Solanum lycopersicum*	5	2	62,739	6.56	Metabolism
B7	Q8LKB0	enolase	*Musa acuminata*	21	2	15,864	7.83	Metabolism
B8	Q06H19	UDP glucose pyrophosphorylase	*Arachis hypogaea*	22	3	16,851	7.30	Metabolism
B9	Q8S9B8	UGPase PC	*Pyrus pyrifolia*	3	1	50,719	5.90	Metabolism
B10	B2X0E6	glyceraldehyde 3 phosphate dehydrogenase	*Mallotus nesophilus*	47	2	32,795	5.96	Metabolism
B11	Q5PY03	glyceraldehyde 3 phosphate dehydrogenase	*Musa acuminata*	2	1	35,974	6.20	Metabolism
B12	Q2XQF4	glyceraldehyde 3 phosphate dehydrogenase	*Elaeis guineensis*	8	2	32,135	7.42	Metabolism
B13	P34922	glyceraldehyde 3 phosphate dehydrogenase	*Pisum sativum*	9	3	36,586	6.63	Metabolism
B14	A5JEJ7	glyceraldehyde 3 phosphate dehydrogenase	*Zehneria keayana*	13	1	7,001	9.87	Metabolism
B15	P84733	Putative cytochrome c oxidase subunit II PS17	*Pinus strobus*	50	1	33,265	7.42	Unknown
B16	O82450	branched chain alpha keto acid decarboxylase	*Arabidopsis thaliana*	2	1	38,709	6.27	Oxidoreductase
B17	Q5ILG5	cysteine protease gp3a	*Zingiber officinale*	4	4	52,062	6.17	Peptidase
B18	Q01H20	Predicted ATPase (ISS)	*Ostreococcus tauri*	1	1	57,490	5.84	ATP binding
B19	P25251	cysteine protease COT44	*Brassica napus*	4	1	36,277	8.05	Peptidase
B21	Q9FE01	L-ascorbate peroxidase 2	*Oryza sativa*	15	3	27,101	5.21	Stress/Defense
B23	Q41561	Heat shock protein 16.9C	*Triticum aestivum*	16	2	14,376	6.23	Stress
B24	O22373	Superoxide dismutase [Cu-Zn]	*Capsicum annuum*	10	2	15,279	5.13	Stress/Defense
B33	Q8LEA2	Gibberellin 2-beta-dioxygenase 1	*Arabidopsis thaliana*	7	2	36,709	8.53	Oxidoreductase
B34	Q94A43	BES1/BZR1 homolog protein 2	*Arabidopsis thaliana*	8	1	34,174	8.63	Transcription
B37	Q09023	Endochitinase CH25 precursor	*Brassica napus*	8	1	34,793	6.29	Metabolism

In the acidic region, three mannose-binding lectins with an observed mass range of 14.4 - 17 kDa (spots A20, A27 and A34) were found. Note, however, that the predicted (theoretical) mass of the homologous proteins used to identify these three spots are slightly higher for A27 and A34 (19.5 and 20.3 kDa, respectively) but significantly so for A20 with a predicted mass of 404 kDa. A mannose binding lectin with a molecular mass of 13.4 kDa was also isolated from *C. zedoary *[39]. In addition, six homologous lectin proteins of various molecular masses (8.84-32.8 kDa) were found in *C. aromatica *[40]. Most of them are mannose binding lectins. With respect to high throughput protein identification, agglutinin was also found to be present in the *C. longa *2-D IEF-SDS-PAGE protein profile [33] at around 14.4 kDa in the acidic region (pI~4.6) which is similar to spot 27 here.

Eleven of the putatively identified proteins from the basic region (Figure 2B) in the 2-D protein pattern were likely to be involved in plant metabolism. Enolase, a ubiquitous enzyme that catalyzes the conversion of 2-phosphoglycerate to phosphoenolpyruvate in the glycolytic pathway, was identified as spot B7. Endochitinase, an enzyme that belongs to the glycosyl hydrolase family and is involved in carbohydrate metabolism and chitin degradation, was present in spot B37. Glyceraldehyde 3 phosphate dehydrogenase, an enzyme that catalyzes the conversion of glyceraldehyde 3 phosphate to D-glycerate 1,3-bisphosphate in the sixth step of glycolysis, was identified as spots B10-14. Four other glycogenesis proteins, phosphoglucomutase, glucose-6-phosphate isomerase, UDP glucose pyrophosphorylase and UGPase, were identified in spots B4, B6, B8 and B9 respectively. Interestingly, two antioxidant proteins were found in the basic region. Superoxide dismutase (SOD), a class of enzymes that convert the reactive superoxide radical into oxygen and hydrogen peroxide, was identified in spot B24. This result is in accord with the recent report of an antioxidant activity and the isolation of a SOD homologue from *C. comosa *[41]. Indeed, SOD homologues have also been reported in other Zingiberaceae plant species, such as *C. longa *[12] and *C. zedoaria *Roscoe [13]. Their current biotechnological application has mainly been in cosmetic products to reduce free radical levels that otherwise cause skin damage [42]. Ascorbate peroxidase, an enzyme that detoxifies peroxides by using ascorbate as the substrate, was found as spot B21. The main function of this enzyme is control the hydrogen peroxide concentration in cells. The discovery of these two antioxidant enzymes may suggest some benefit for *C. comosa *for the natural product based cosmetic industry, but this will depend upon their relative specific activity or ease of enrichment. Moreover putative cysteine proteases were identified as spots B17 and B19 at molecular weigh about 20.1 kDa and 14.4 kDa respectively. This enzyme family plays a role in plant growth, development and senescence. Most plant cysteine proteases belong to the papain and legumain families. Recently this enzyme family was reported from three members of the ginger family, in *C. longa *[43], *C. aromatica *[40] and *Z. offinale *Roscoe [44], and this ginger protease is used as a food improver and anti-inflammatory agent. Founding cysteine protease in four members of Zingiberaceae plant, *C. comosa, C. longa, C. aromatica *and *Z. offinale *at difference molecular weigh and pI position, the ginger cysteine protease might be a protein marker to classify specific species in this family in the future.

## Conclusion

The protein profile of *C. comosa *was improved by separation by microscale solution-phase isoelectrofocusing, and identified in part by using high throughput two-dimensional IEF-SDS-PAGE together with tandem mass spectrometry. Some proteins were identified as lectins and antioxidant proteins, which appears to be related with their activity and cysteine proteases that are also found in other Zingiberaceae plant species.

## Competing interests

The authors declare that they have no competing interests.

## Authors' contributions

BA carried out the whole project experiment and drafted the manuscript. SR participated in the design of the study and mass spectroscopy and drafted the manuscript. CD carried out in mass spectroscopy. KA participated in drafted the manuscript and coordination. SP participated in the design of the study and mass spectroscopy and drafted the manuscript. All authors read and approved the final manuscript.
